# Assessing the Impact of Heat Treatment of Food on Antimicrobial Resistance Genes and Their Potential Uptake by Other Bacteria—A Critical Review

**DOI:** 10.3390/antibiotics10121440

**Published:** 2021-11-24

**Authors:** Christian James, Ronald Dixon, Luke Talbot, Stephen J. James, Nicola Williams, Bukola A. Onarinde

**Affiliations:** 1 Food Refrigeration & Process Engineering Research Centre (FRPERC), Grimsby Institute, Nuns Corner, Grimsby DN34 5BQ, UK; talbotl@grimsby.ac.uk (L.T.); jamess@grimsby.ac.uk (S.J.J.); 2 National Centre for Food Manufacturing (NCFM), University of Lincoln, Park Road, Holbeach PE12 7PT, UK; bonarinde@lincoln.ac.uk; 3 Joseph Banks Laboratories, School of Life Sciences, University of Lincoln, Lincoln LN6 7DL, UK; rdixon@lincoln.ac.uk; 4 Institute of Infection, Veterinary and Ecological Sciences, Leahurst Campus, University of Liverpool, Neston CH64 7TE, UK; njwillms@liverpool.ac.uk

**Keywords:** antimicrobial resistance, antimicrobial resistance gene, bacteriophage, food, gene transfer, heat treatment, membrane vesicles, plasmid

## Abstract

The dissemination of antibiotic resistance genes (ARGs) is a global health concern. This study identifies and critically reviews the published evidence on whether cooking (heating) food to eliminate bacterial contamination induces sufficient damage to the functionality of ARGs. Overall, the review found that there is evidence in the literature that Antimicrobial Resistant (AMR) bacteria are no more heat resistant than non-AMR bacteria. Consequently, recommended heat treatments sufficient to kill non-AMR bacteria in food (70 °C for at least 2 min, or equivalent) should be equally effective in killing AMR bacteria. The literature shows there are several mechanisms through which functional genes from AMR bacteria could theoretically persist in heat-treated food and be transferred to other bacteria. The literature search found sparce published evidence on whether ARGs may actually persist in food after effective heat treatments, and whether functional genes can be transferred to other bacteria. However, three publications have demonstrated that functional ARGs in plasmids may be capable of persisting in foods after effective heat treatments. Given the global impact of AMR, there is clearly a need for further practical research on this topic to provide sufficient evidence to fully assess whether there is a risk to human health from the persistence of functional ARGs in heat-treated and cooked foods.

## 1. Introduction

Antimicrobial resistant (AMR) microorganisms and antibiotic resistance genes (ARGs) are a major public health issue globally. It is estimated that unless action is taken to tackle AMR the global impact of AMR could be 10 million deaths annually by 2050 and cost up to USD 100 trillion in cumulative lost economic output [1].

**Figure 1:**
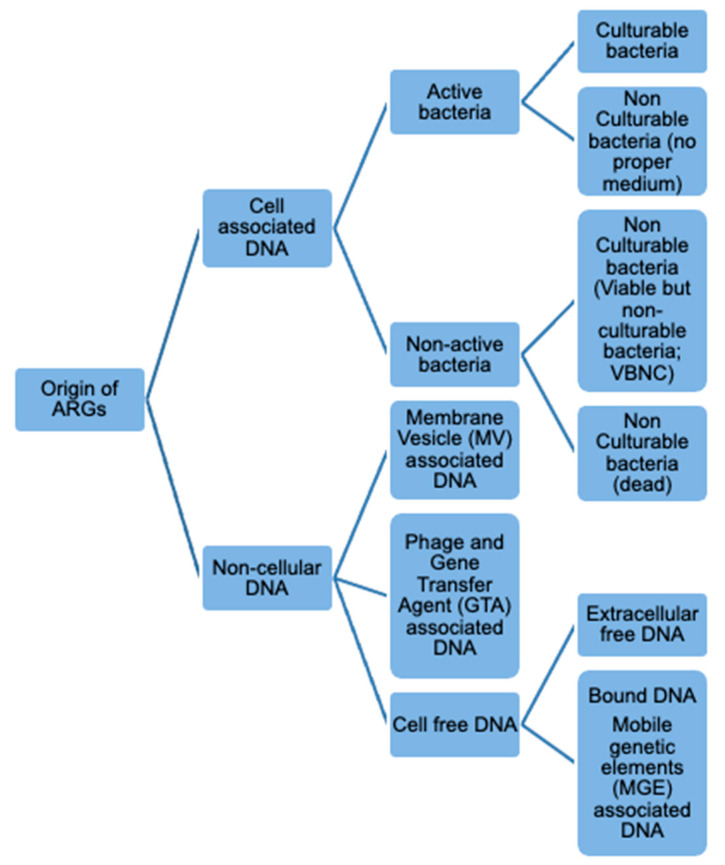
Forms and origins of ARGs quantified by molecular biology approaches.

AMR mechanisms in bacteria may be intrinsic or acquired. ‘Intrinsically resistant’ microorganisms are inherently resistant to antimicrobials [2], whereas AMR can also be acquired either due to mutation (e.g., genomic point mutations), or the acquisition of ARGs by horizontal gene transfer (HGT) [2,3]. Thus, commensal non-pathogenic AMR bacteria can act as a reservoir for ARGs and transfer resistance to non-resistant human or animal pathogenic bacteria [4]. ARGS may be mobilized between bacteria through a wide range of mechanisms some of which do not require live cultivable bacteria (Figure 1). Functional cell-free ARGs, which also cover genes encapsulated in membrane vesicles (MVs), bacteriophages and Gene Transfer Agents (GTAs), can potentially persist after treatments that kill AMR bacteria and transfer to recipient bacteria in the absence of a live donor bacteria [5]. The persistence of functional ARGs in processed food is of growing concern.

There are a number of HGT mechanisms through which ARGs from heat-treated bacteria could be transferred to other bacteria. HGT is enhanced by mobile genetic elements (MGEs), such as plasmids, integrons, and transposons that facilitate the movements of genes [6]. The frequency of HGT largely depends on the properties of the MGE, the characteristics of the donor and recipient populations, and the environment [2,7]. There are three main canonical mechanisms of HGT through which this can occur: (1) conjugation, (2) transformation, or (3) transduction. Although, other less well recognized mechanisms of DNA transfer may occur [2,8].

Conjugation occurs between live bacterial cells and will not occur if cells are killed by heat [2]. Thus, this important mechanism is not relevant in the context of this review and will not be discussed.

DNA fragments, including ARGs, may be released after death and lysis of heat-treated bacteria and be transferred by transformation [2,9,10,11]. Transformation of genes from heat-killed cells was first demonstrated in 1928 [12,13]. Theoretically any bacterial chromosomal or extra-chromosomal DNA can be transferred by transformation [2]. In order to be stabilized in the recipient cell, it is reported that the transformed DNA must be available as a plasmid or must recombine with homologous regions in the resident chromosome [2]. The mechanism of transformation has been described in numerous publications [12,14]. Natural transformation is known to occur in more than 60 bacterial species, and probably far more [7]. However, the consensus in the literature appears to be that the process of transformation occurs at low frequency and is subject to a large number of requirements that are mostly observed in very controlled laboratory conditions [2,12,15]. Few investigations have expressly analyzed exogenous DNA uptake by bacteria in food [7]. There is evidence that DNA stability is an inverse function of DNA length, and that although heat treatments will degrade lysed naked ARGs, fragments may still be of sufficient length to be transformed by other bacteria [16].

Transduction is an HGT mechanism mediated by bacteriophage (phage) and related particles, called gene transfer agents (GTAs) [2]. Phages are capable of packaging part of their host’s genetic material (including ARGs) either by reproducing within the host cell before lysing the cell (lytic) or through incorporation into the host cell genome (lysogenic). The mechanism of how bacteriophages/GTAs promote the transfer of ARGs is described in numerous publications (for example, [17,18,19]).

There is a growing concern that phages/GTAs may be significant vectors in the transmission of ARGs, although there is debate on their importance [18,19]. Although phages have been believed to be narrow host-specific, there is growing evidence that phages can have broader host ranges [19]. The occurrence of phages/GTAs harboring ARGs has been reported in different food and animal matrices [19,20]. Notably ARGs have been detected in DNA extracted from phage particles extracted from retailed ready-to-eat (RTE) samples of cooked ham (*bla*_TEM_, *bla*_CTX-M-1_, *bla*_CTX-M-9_, *bla*_OXA-48_, *bla*_VIM_, and *sul*1) and in mortadella (*sul*1) [20]. Though it must be stressed that the study did not associate this presence with survival following thermal processing, but rather post-processing cross-contamination. Nevertheless, the survival of phage containing ARGs following heat treatment is theoretically possible and cannot be ruled out.

Phages can show a degree of thermal stability and in some cases may survive heat treatments that are sufficient to kill target bacteria [21,22,23]. The survival of thermal-stable lactococcal phages in pasteurized milk is a long-recognized problem [24]. It has been theorized that phages harboring ARGs could survive processing treatments in RTE foods and these ARGs could be transduced to host bacteria occurring in the human gut when consumed [19].

A further mechanism that is receiving increasing attention is membrane vesicle (MV)-mediated HGT [5]. MVs are proteo-liposomal nanoparticles produced by both Gram-negative and Gram-positive bacteria generally in response to environmental stresses and exposure to antibiotics [25]. They have diverse functions, including the transport of virulence factors, DNA transfer (including ARGs), interception of bacteriophages, antibiotics and eukaryotic host defense factors, cell detoxification and bacterial communication [26]. MVs were first found to originate in the outer membrane of Gram-negative bacteria and are therefore often called outer-membrane vesicles (OMVs); however, recent work has shown that different types of MVs also exist and hence the inclusive term MV is preferred [5,27] and used in this review. Studies have found the presence of DNA of chromosomal, plasmid, and phage origin incorporated into MVs [27]. MVs have been found to transfer ARGs between bacterial species [27,28,29]. The occurrence of MV-mediated HGT in the environment has been largely unexplored [5].

The literature shows that HGT mechanisms clearly exist that could facilitate the survival and transfer of functional ARGs from heat-treated bacteria to surrounding viable bacteria present, including the human gut and foods. The focus of this review, commissioned by the UK Food Standards Agency, was to assess what evidence exists on the impact of heat treatments on ARGs that may be present in heat-killed foodborne bacteria and their potential uptake by other ‘live’ bacteria in the human gut and foods. A full report of this project is available from the UK Food Standards Agency [30].

## 2. Results

Following the literature search, a total of 2681 articles were initially screened. Of these, a total of 247 publications were considered relevant by title and abstract and full texts collected for screening; this was reduced to 53 publications from which some data were extracted. Of these publications, 9 publications were reviews with some mention of the impact of heat on AMR bacteria, while 17 publications had evidence on the relative heat resistance of AMR bacteria in comparison to non-AMR bacteria. However, only four publications were identified that were considered to fully meet the search criteria, i.e., had considered the impact of heat treatments on the persistence of ARGs after such treatments.

## 3. Discussion

The literature search identified nine publications in the last decade that in part reviewed aspects of the thermal resistance of AMR bacteria in foods subjected to heat treatments [2,11,31,32,33,34,35,36,37]. The survival of AMR bacteria in insufficiently heat-treated foods, and whether AMR bacteria are more heat resistant than non-AMR bacteria are discussed in part in some of these reviews [33,34]. Three mention the theoretical persistence of ARGs either within intact dead cells or from lysed cells after heat treatment [2,11,33]. However, none of these reviews provide any citations that have demonstrated this or discuss this in any depth. One review includes a comprehensive review of the heat tolerance of AMR bacteria, but does not consider the impact on, or persistence of, ARGs after any heat treatment [34]. Overall, the consensus of these reviews is that: (1) heat treatments capable of eliminating non-AMR bacteria are equally effective in eliminating AMR bacteria; (2) the presence of AMR bacteria or genes in cooked food after cooking is likely to be the result of insufficient heat treatment or contamination after cooking.

A comprehensive review of the impact of food processing on AMR bacteria in secondary processed meats and meat products found no specific publications describing the fate of AMR bacteria or ARGs after thermal processing [36]. The authors note that there are reports of AMR bacteria still being isolated from cooked meats following processing (and we would also note, ARGs) [38,39,40,41,42]. These surveys, collected at retail and in foodservice, did not determine if the AMR bacteria/genes were detected after effective cooking or were due to post-process contamination. Similar surveys of pasteurized and sterilized milk report the presence of AMR bacteria/genes, but again this may also be due to post-treatment contamination [43]. In addition, as previously discussed in the introduction, ARGs have been detected in DNA extracted from phage particles extracted from cooked ham and mortadella [20]. These studies (with one exception) do not appear to have considered the possibility that ARGs could have persisted following effective thermal processing. The study of pasteurized and sterilized milk did report evidence that heat-treated bacteria could have been in a viable but non-culturable (VBNC) and metabolically active and able to transcribe genes [43].

### 3.1. Heat Resistance of AMR Bacteria

It is accepted in the literature that heat treatments such as sterilization, ultra-high temperature (UHT) treatment, and (full, traditional) pasteurization under well-defined time/temperature combinations will kill vegetative bacterial cells, including AMR bacteria. Industrial, food service, domestic or institutional cooking undertaken correctly is normally sufficient to eliminate bacterial pathogens from food.

Studies have indicated that foodborne AMR bacteria, such as *Escherichia coli*, *Listeria monocytogenes*, *Salmonella* spp., *Staphylococcus aureus*, *Yersinia enterocolitica*, do not exhibit enhanced thermal resistance characteristics (Table 1). Two studies on AMR serotypes of *Salmonella* concluded that there was no evidence of any association between antimicrobial susceptibility and the ability of AMR serotypes to survive or repair damage associated with heat stress [44,45]. Some studies provide evidence that AMR may impair thermal tolerance in bacteria [46,47,48].

The majority of published reviews and studies conclude that there is no evidence to suggest that AMR bacteria are more heat tolerant than non-AMR bacteria, with the exception of three studies [51,60,61].

One study has reported that *S. enterica* Typhimurium definitive phage type (DT) 104 (= *S*. Typhimurium DT 104), an AMR strain, was more heat resistant (based on D-values at 55 °C) than non-AMR strains [51]. This study also found that *S*. Typhimurium DT 104 subject to a sub-lethal heat shock (48 °C for 30 min) was significantly more heat resistant than non-heat-shocked *S.* Typhimurium DT104, indicating that heat shocking-conferred thermo-tolerance could be incited in this strain. It must be stressed that conferred thermotolerance is not unique to this strain or related to AMR. A study on the impact of a dry heat treatment at 70 °C for up to an hour on inoculated strains of *Salmonella* spp. on beef, lamb, and goat meat in the context of a processing CCP intervention step also reported AMR strains to be particularly heat resistant [61]. Of the *Salmonella* strains used, an AMR strain of *S*. Typhimurium 2470 on beef and lamb, and AMR strains of *S*. Heidelberg (329 and 2581) on lamb were more heat resistant than other strains. A study of heat resistant MRSA isolated from pasteurized camel milk found that a high proportion (10%) of MRSA were more heat resistant than a reference strain of *S. aureus* [60]. In contrast, other studies have found MRSA in heat-treated milk to be less heat tolerant to methicillin-susceptible *S. aureus* (MSSA) [53].

Few publications have compared the efficacy of heat treatments used as interventions during the processing of red meat and poultry (as used in the US) on AMR, or susceptible bacteria. AMR strains were reported to be no more heat resistant than non-AMR strains, in relation to Salmonella in beef [62]. Though as previously reported, AMR strains of *Salmonella* were more heat resistant than non-AMR strains to a dry heat treatment [61].

There would appear to be little specific data on the impact of commercial thermal processing on AMR bacteria in foods. The literature search only identified one publication on the impact of dielectric heating (microwave or radio frequency (RF)). The use of nalidixic acid resistant strains of three major Shiga toxin-producing *E. coli* (STEC) and non-pathogenic *E. coli* for use as marker organisms to challenge test the effectiveness of RF heat treatments was evaluated [56]. They concluded that the heat resistance of nalidixic acid-resistant strains were not significantly different to nalidixic sensitive strains at the endpoint temperatures investigated (55, 60, and 65 °C).

While there are many publications on the thermal inactivation of bacteria (particularly pathogens) during a wide variety of cooking operations, especially regarding the gridling of burgers (patties) and steaks, the literature search identified no publications on the possible impact of different domestic or foodservice cooking methods specifically on AMR bacteria.

That said, laboratory-acquired AMR strains of bacteria are often used as “marker” strains for use in process validation experiments [52]. Prior to their use, their heat resistance is usually compared with non-AMR strains to establish their fitness for such purpose and that their heat resistance is similar to but not greater than the target organism [63,64,65]. Thus, there is a wealth of published and laboratory data that do demonstrate that the heat resistance characteristics of laboratory-acquired AMR bacteria are similar to their parent strains.

A number of publications note that increased use of sublethal, rather than lethal, food preservation heat treatments may be more important than was previously considered for the development and dissemination of AMR bacteria and genes [2,11,35,66,67]. They note that mild heat treatments (45–60 °C) may be ineffective in inactivating both AMR and non-AMR microorganisms and could trigger bacterial stress responses. However, none of these publications cite published evidence of what impact “mild heat treatments” could have on ARGs and the literature search did not identify further clear evidence on this risk.

There is evidence that stress conditions (such as heat stress) may trigger several mechanisms in bacterial cells, e.g., stress adaptation, cellular repair, application of response mechanisms and enhanced virulence [68]. In their review of sublethal injury, Wesche et al. [68] noted that thermal treatments that included an extended “come-up phase”, such as slow roasting of meats, or certain sous-vide processes, might cause sublethal injury to microorganisms. It has been reported that incubation at a sub-lethal temperature (45 °C) increased the antimicrobial susceptibility of strains of *E. coli, S.* Typhimurium DT 104, and *S. aureus* [9]. While heat shocking (48 °C for 30 min) has been shown to confer thermotolerance in *S*. Typhimurium DT 104 [52]. However, there appears to be little other published evidence on this subject in the literature.

Overall, the literature provides clear evidence, as may be expected, that there are differences in thermal tolerance between different bacteria species, serotypes, or strains, and different substrates, whether the bacteria are AMR or non-AMR. However, none of these publications on the comparative heat resistance of AMR bacteria provide evidence of whether functional ARGs may survive such heat treatments or consider whether ARGs may survive the heat processes applied.

### 3.2. Fate of ARGs in Heat-Treated Food

Heat will denature, degrade, and fragment DNA. It is fully accepted that heat treatments such as sterilization, UHT treatment, and pasteurization under well-defined time/temperature combinations will eradicate/kill vegetative bacterial cells and other microorganisms, including AMR bacteria. That in part is due to damage to their DNA, though no single event is responsible for cell death [69].

There is evidence, however, that bacterial DNA is not denatured by some heat treatments that would be expected to be sufficient to kill bacteria. Examination of the thermal denaturation of bacterial cells by differential scanning calorimetry (DSC) has shown that higher temperatures are needed to denature DNA than kill bacterial cells [70,71]. Fragments of bacterial DNA (part of the eaeA gene of *E. coli* O157:H7) have been reported to not be denatured when heated at 95 °C for up to 30 min [72]. It has also been reported that microbially derived DNA is still capable of being amplified by PCR when treated at 100 °C for up to 240 min [73]. However, these studies did not study the functionality of the DNA. PCR detection does not prove that such genes are functional, either because the DNA integrity has been breached or regulatory proteins have coagulated. However, there is evidence that, although heat treatment does degrade lysed extracellular free DNA, surviving fragments may still be still of sufficient integrity to be transformed by other bacteria [16].

The literature search identified only four publications that have directly addressed the fate of ARGs in heat-treated foods (Table 2). Of these, only one specifically looked at the fate of ARGs in conventionally cooked food in an in vitro mimic of cooking processes [74]. Of the other publications, one was an in vitro mimic of commercial milk pasteurization [75]; another was in vitro and not designed to mimic any particular heat treatment but did use strains originating from animal sources and temperatures and time similar to thermal processes used to treat and cook food [10]. A further publication that was considered relevant, but had not been applied to food, was an in vitro mimic of autoclaving [76].

Koncan et al. [74] studied whether *aac*(6′)-*aph*(2′)-modifying aminoglycoside ARG could be detected in meat (chicken, pork, and beef) after conventional cooking procedures. This gene is reported to be encoded by plasmids and transposons, to be widely spread in *E. faecalis*, and confers resistance to most available aminoglycosides, except to streptomycin [77]. Food samples were either boiled (20 min), grilled on a cooking plate (10 min), microwaved (5 min, 900 W), or autoclaved for 20 min at 1 atmosphere and 121 °C. After all of the heat treatments no bacteria were detected but positive PCR results for the bifunctional gene were observed in all samples. A direct correlation between the density of bacterial inoculum and the intensity of amplified DNA was also observed. Differences between medium were also found, with higher amounts of the bifunctional gene recovered in the beef samples than in the pork or chicken. Transformation experiments to recipient *E. faecalis* JH2-2 with total DNA from samples were negative in all cases. Though lacking in detail and presented as a poster, this is the only study identified that has addressed the fate of ARGs in conventionally cooked food. This study does suggest that heat treatments that are capable of killing bacteria may not fully destroy ARGs. It also suggests that the survival of ARGs in foods subjected to the same heat treatment is different in different food matrices (which may be expected as the type of food matrix is known to contribute to the heat resistance of bacteria, [78,79]; it is likely that this is due to differences in the thermophysical and structural properties of different foods). This study did not provide any evidence that the gene that was detected following heat treatment was a functional gene. Since DNA remaining after heat treatment is likely to be highly fragmented, a PCR test will still detect highly fragmented DNA remaining after heat treatment and produce a PCR positive amplicon. That the study was unable to transfer genes to a competent recipient strain may indicate that the genes were indeed not functional.

In another study, the impact of heat treatment, using a traditional water bath method, on the possibility of ARGs being transferred from extended spectrum beta-lactamase (ESBL) *E. coli* cells was evaluated [10]. Treatment at 60 °C for 20 min and longer, and all treatments at 70 °C, reduced bacterial numbers to below the limit of detection. However, PCR analysis identified amplicons of the *bla*_CTX-M_, *bla*_CMY-2_, *tetA* or *strA* ARGs in heat-treated suspensions. Transformation assays (by electroporation) using suspensions heated to 70 °C for 30 min, from which no bacteria could be isolated, demonstrated that genes coding for resistance to extended-spectrum cephalosporins, tetracycline or sulfonamides carried on a conjunctive plasmid could be transferred to an *E. coli* DH5α recipient. Although, only a limited number of positive results were obtained, in 2 out of 12 trials, indicating that its occurrence is probably rare. Additionally, as the study’s authors point out, the numbers in original suspensions were very high, indicating that a great number of AMR bacteria may need to be present as contaminants for sufficient ARGs to persist after heat treatment for any transfer to other bacteria to take place.

It was concluded in the study that the heat-treated bacteria had been inactivated since transformant colonies could be detected after plating [10]. However, the authors highlighted that they could not exclude the possibility that suspensions still contained a few viable cells. We would suggest that another possibility was that heat-treated bacteria could have been in a VBNC state. Studies suggests that AMR bacteria in a VBNC state are metabolically active and able to transcribe and translate genes [43,75,80].

Taher et al. [75] reported that a standard milk pasteurization treatment (63.5 °C for 30 min) was not sufficient to inactivate plasmid-mediated ARGs (*blaZ*, *mecC*, and *tetK*) of staphylococci (*S. aureus* and *S. sciuri*) and, in addition, would induce a VBNC state in these bacteria. In this study, milk and elution buffer were spiked at levels of 10^5^ and 10^6^ organisms, pasteurized (63.5 °C for 30 min) or sterilized (121 °C for 15 min), and stored for up to 21 days at 4 °C. Copy numbers of the genes were quantified through PCR and qPCR after the heat treatments and during storage. Copy numbers of *blaZ* (which encodes for penicillin resistance), and tetK (which encodes for tetracycline resistance) genes remained similar after pasteurization, while numbers of the mecC (which encodes for methicillin resistance) genes were lower, however all increased over time. Cultivability tests were negative, however use of the BacLight LIVE/DEAD stain showed a significant number of ‘live’ (green fluorescent) microorganisms in the pasteurized samples; qPCR of 16S ribosomal DNA was also used to quantify VBNC. To assess whether the tested genes were still active, expressed, and if resistance was still transferable to another microorganism, detection of the transmissibility of the tested genes was conducted in vitro using the electro-competent *S. aureus* RN42200 strain. The recipient cells showed resistance to methicillin and tetracycline after transformation using electroporation, thus indicating that both mecC and tetK ARGs were still functional and able to be expressed.

Overall, this study provides evidence that AMR bacteria may persist in a VBNC state in heat-treated foods and that ARGs from these heat-treated bacteria may be still expressed and transferable. The occurrence of gene expression (though not ARGs) by VBNC bacteria after milk pasteurization (63.5 °C for 30 min, the same treatment that Taher et al. [75] used) has also been reported in another study [80].

The only other publication identified in the literature search as partially relevant, though not applied to food, was an in vitro mimic of autoclaving [76]. This demonstrated that a laboratory constructed plasmid (pUC18) heated in distilled water at 121 °C for 15 min in the presence of 0.5–2.0 mL L^−1^ sodium chloride was still capable of transforming ampicillin resistance to *E. coli* (DH5 α) by electroporation. No transformable activity was detected however when a plasmid preparation was autoclaved at 135 °C for 20 min. No further studies appear to have been carried out on this subject by these researchers. The implications of these findings in relation to the persistence of functional ARGs in heat-treated foods do not appear to have been further studied by other researchers and this publication has not been cited by any other publication on this specific topic (i.e., heat resistance of ARGs). Since many cooked foods contain sodium chloride and receive a far less severe heat treatment than that studied, this study provides some interesting preliminary evidence that functional ARGs in plasmids may be capable of persisting in foods after heat treatments.

It must be noted that all four studies used electroporation to assess transformability. One study concluded that, while the possibility of ARGs being transferred from heat-inactivated *E. coli* via natural transformation during food preparation could not be excluded, it was likely to be infrequent [10]. In addition, only two of the studies used food matrices [74,75], the other two [10,76] used simple saline matrices. It is highly likely that the heat resistance of bacteria and ARGs in complex media or food matrices will differ from that in simple matrices [10,78,79].

No publications were identified that have directly compared the behavior of chromosomal DNA and plasmid DNA in response to heat. The four studies that were identified appear to have considered only their survival in plasmid-mediated DNA, though the resistances could be both plasmid and chromosomal. While one of the four did not specifically mention plasmids, the gene they investigated, *aac(6′)-Ie-aph(2″)-Ia*, is reported to be encoded by plasmids and transposons [77].

While the literature suggests that heat-tolerant phages/GTAs and MVs could potentially be important vectors in the transfer of ARGs in heat-treated food, the literature search found no published studies that have addressed these topics. The mechanisms responsible for phage/GTA and MV transfer of ARGs and their importance and role in the transfer of ARGs do not yet appear to have been fully explored. There currently appears to be little evidence of whether these vectors are more than a theoretical risk. It has been reported that heat-treated MVs from *S. aureus* (ATCC 14458) containing *blaZ*, a β-lactamase protein, do not mediate the survival of ampicillin-susceptible bacteria [81]. This study did not find *blaZ* genes in MVs from *S. aureus* but did identify MVs containing the βeta-lactamase protein. They found that non-heat-treated MVs containing this protein did enable other ampicillin-susceptible Gram-negative and Gram-positive bacteria to survive in the presence of ampicillin. However, MVs that contained this protein that were first heated to 100 °C for 20 min did not mediate the survival of ampicillin-susceptible bacteria in the presence of ampicillin. This provides some evidence that high temperature heat treatments may inactivate ARGs in MVs. However, the heat treatment used was at a much higher temperature and longer time than a food heat treatment equivalent to 70 °C for at least 2 min, and the MVs were in a simple saline matrix rather than a complex food matrix.

Different heat treatments, and heating rates [79], are highly likely to have an impact on the survival and viability ARGs, whether as cell free DNA or in mobile elements such as phage/GTAs or MVs. Only one of the studies identified studied different cooking treatments, however these treatments were limited, and no time-temperatures were provided [74]. It is likely that the most important factors will be the maximum temperature the gene is subjected to, the duration at this temperature and temperature history (come-up and come-down times), and the type of food matrix.

### 3.3. Transfer of ARGs in the Human Gut from Heat-Treated Food

Theoretically, ARGs that are not destroyed during heat treatment and passing through stomach acid may be capable of transfer to other microbiota in the human gut and be incorporated, thereby becoming a functional source of AMR.

There are some studies [82,83,84] that lend weight to this hypothesis, although the literature search identified no evidence of ARGs from heat-treated or cooked food being shown to transfer to other microbiota in the human gut. Reviews of ARG exchange in the gut have been carried out (such as [14,85,86,87,88]) but whether ARGs from heat-treated foods can be a source of transfer is not discussed in these reviews.

It is clear, that the capacity for the acquisition of ARGs by gut microbiota deserves more intensive study [43,75,89]. In addition to the human gut environment, some studies exist to indicate that the food environment could potentially facilitate uptake of DNA by certain bacteria [90].

## 4. Materials and Methods

A systematic review approach was taken to the literature search; however, owing to the identification of a lack of specific published studies on this topic, a narrative critical review approach was taken to the review of the publications identified.

The review question was:

“Do different heat treatments applied to eliminate bacterial contamination in foods also induce sufficient damage to ARGs to prevent or inhibit their uptake by surrounding viable bacteria present in other settings, including the human gut and foods?”

The review adopted a comprehensive search strategy considering all available evidence in the public domain, including peer-reviewed articles, grey literature (e.g., government and industry reports), relevant government reports (e.g., FSA published studies, ACMSF reports, etc.), European and International literature (e.g., the EFSA Scientific Opinions, WHO reports) up to December 2020. This included previously published systematic and critical reviews, and risk assessments, as well as primary research.

The primary source databases searched were Web of Science, and PubMed. The searches were restricted to records published from 1990 to present. Finalized keywords were agreed with the Agency and were:

Antimicrobial resistance OR antimicrobial resistant OR antibiotic resistance OR antibiotic resistant OR antibacterial resistance OR antibacterial resistant OR drug resistant OR multi resistance OR multi resistant OR multidrug resistance OR multidrug resistant OR multi-drug resistance OR multi-drug resistant OR multiantibiotic resistance OR multiantibiotic resistant OR AMR OR MDR OR MAR OR AR OR AMRG

And

Acinetobacter OR Campylobacter OR commensal OR Enterobacter OR Enterococcus OR Escherichia coli OR E. coli OR Klebsiella OR Listeria OR Salmonella OR Staphylococcus OR pathogen OR Pseudomonas

And

Blanch* OR boil* OR canning OR cook* OR fried OR fry* OR griddle OR grill* OR heat OR “high temperature” OR HTST OR “hot fat” OR “hot fat” OR “hot oil” OR “hot water” OR microwave* OR oven OR pasteuri* OR “pressure cook*” OR retorting OR roast OR “sous vide” OR steam OR steili* OR thermal OR UHT

Focused Google searches were used to identify relevant grey literature. In total, 2446 citations were initially identified in Web of Science and 937 were identified in PubMed. There was some overlap between the databases with 737 duplicates. An additional 35 records were identified through Google searches, other references, and through contact with authors. For all searches, citations and abstracts were uploaded from each of the electronic databases into Covidence. The following exclusion criteria were applied:

(1) The publication did not address the impact of heat treatments on AMR bacteria or genes;
(2) The publication was in a language other than English;
(3) The publication measured irrelevant interventions (no heat treatment), outcomes, or populations or samples.

The criteria were independently applied to the abstract of each paper by at least two members of the five-member project team. For each citation, a consensus was reached that the citation is relevant for inclusion. Arbitration by a third member of the project team was used to settle conflicting appraisals. Full texts were obtained for all abstracts that passed the inclusion criteria.

## 5. Conclusions

This literature review has shown that there is evidence that AMR bacteria are likely to be no more heat resistant than non-AMR bacteria and that there is therefore evidence that heat treatments sufficient to kill non-AMR bacteria (such as 70 °C at least 2 min, or the equivalent) will be equally effective in killing AMR bacteria. However, there is sparse published evidence on whether functional ARGs may persist in food after such heat treatments, and whether these functional genes could be transferred to other bacteria. Currently, there is a paucity of evidence to determine if there is a risk.

The literature suggests that theoretically functional ARGs could potentially survive in heat-treated food either as (1) naked DNA lysed from heat-treated AMR bacterial cells, (2) within heat tolerant phage (and related particles), (3) within MVs, (4) within VBNC heat-treated AMR bacterial cells. These genes could, subsequently, theoretically, be transferred to live bacteria through a range of HGT mechanisms.

It is clear that there are insufficient numbers of published studies on this subject to carry out any reliable analysis of the data or draw reliable conclusions regarding the evidence on the impact of different heat treatments on ARG uptake by viable bacteria.

What limited evidence exists does imply that heat treatments that are effective at eliminating bacteria may not be sufficient to destroy ARGs, that these genes could remain functional, and that it is possible in the laboratory to transfer those genes to other bacteria. We caution that some of the evidence of survival of ARGs after heat treatment is limited to positive results with PCR only and do not therefore provide any proof of gene expression or functionality. No evidence has been found that ARGs from heat-treated food may transfer to bacteria in the human gut after ingestion. Since there is some evidence (though limited) that this potential exists, in our opinion further appropriate practical studies need to be carried out to explore this subject in greater detail. These need to simulate typical heating/cooking cycles in real food matrices to provide reliable and sufficient evidence to demonstrate if there is a risk or not.

## Figures and Tables

**Table 1:** A summary of studies that have compared the heat resistance of antimicrobial resistance (AMR) and non-AMR bacteria.

Evaluation Temperatures (°C)	Medium	Species and Strains	Enhanced Thermal Resistance	Stated Antimicrobial Resistance Profiles (Antimicrobial or Class)	Reference
50–60	Minced beef and potato	*Y. enterocolitica*	No	Nalidixic acid	[46]
47	Oysters	*Vibrio vulnificus*	No	Nalidixic acid	[47]
54, 82	Egg white powder	*S.* Typhimurium DT104	No	NS	[49]
51, 53, 55, 57, 59, 61	Liquid whole egg, egg yolk, egg white, whole egg + 10% salt, egg yolk + 10% salt	*S*. Typhimurium DT104	No	NS, but strains of DT104 quoted as being resistant to ampicillin, chloramphenicol streptomycin, sulfonamides, tetracyclines	[50]
55	Minced beef and potato	*L. monocytogenes*	No	Streptomycin	[51]
55, 57, 59, 61	Tryptic soy broth (TSB)	*Salmonella* spp. serovars Saint-Paul, Anatum, Mbandaka, Agona, Reading, Typhimurium (DT104)	No	Ampicillin, chloramphenicol, streptomycin, sulfonamides, and tetracycline, amoxicillin-clavulanic acid, ampicillin-sulbactam, gentamicin, trimethoprim-sulfamethoxazole Depending on serotype or strain	[45]
55	Chicken pieces	*S.* Typhimurium DT104	Yes	Ampicillin, streptomycin, sulfonamides, chloramphenicol, tetracyclines	[52]
55	Chicken pieces	*S.* Enteritidis, *S.* Typhimurium	No	Nalidixic acid, streptomycin	[52]
55	Minced beef	*E. coli* O157:H7, O26	No	Ampicillin, kanamycin, streptomycin, trimethoprim, nalidixic acid, rifampicin, sulfonamides, chloramphenicol, tetracycline, minocycline, doxycycline Depending on serotype or strain	[48]
55, 60, 65, 70	Tryptic soy broth (TSB)	*Salmonella* spp. serovars Montevideo, Typhimurium, Anatum, Muenster, Newport, Mbandaka, Dublin Reading, Agona, Give	No	Ampicillin, chloramphenicol, streptomycin, sulfonamides, tetracycline, amoxicillin–clavulanic acid, kanamycin, sulfamethoxazole-trimethoprim, gentamicin Depending on serotype or strain	[44]
56	Whole milk	*mecA-* carrying *Staphylococcus* spp. strains (*S. epidermidis*, *haemolyticus, lentus*)	No	Tetracycline, kanamycin, spectinomycin, erythromycin, trimethoprim, sulfamethoxazole-trimethoprim Depending on serotype or strain	[53]
57	Tryptic soy broth (TSB-G)	60 *Salmonella* spp. *serovars including:* Typhimurium (18 strains), Enteritidis (10 strains), Newport (9 strains), Heidelberg (8 strains), Montevideo (4 strains), Senftenberg (4 strains), Agona (3 strains), Infantis (3 strains) and Derby (1 strain).	No	NS	[54]
60, 61, 62.5	Tryptic soy broth (TSB)	*E. coli* (STEC) serotypes O26 and O103	No	Ampicillin, penicillin, ceftiofur, spectinomycin, oxytetracycline, clindamycin, sulfadimethoxime, tiamulin, tilmicosin, tetracycline Depending on serotype or strain	[55]
55, 60, 65 (Radio Frequency heating)	Phosphate buffer saline (PBS)	*E. coli* (STEC) serotypes O157:H7, O26:H11, O11	No	Nalidixic acid	[56]
58	Ringer’s solution	*L. monocytogenes*	No	Erythromycin, ciprofloxacin, nitrofurantoin	[57]
63	Saline solution	*S. aureus*	No	Ciprofloxacin, chloramphenicol, erythromycin, penicillin, sulfamethoxazole, clindamycin, tetracycline, oxacillin, cefoxitin, gentamicin, ciprofloxacin Depending on serotype or strain	[58]
55, 60, 65	Minced chicken	Extraintestinal pathogenic *E. coli* (ExPEC)	No	Aminoglycosides, macrolides, sulfonamides, trimethoprim, tetracycline, beta-lactams, cefotaxime, phenicol, aminoglycosides, streptomycin Depending on serotype or strain	[59]
85, 95	BHI medium	*MRSA* *S. aureus* (ATCC 29,737, control)	Yes	Cefoxitin, cefadroxil, cephalothin, colistin, polymyxin, aminoglycosides, streptomycin, amikacin, kanamycin:cyclic peptides, bacitracin, tetracycline: sulfonamide, sulfamethoxazole, nalidixic acid:fluoroquinolone, ciprofloxacin:oxazolidone, linezolid:macrobid	[60]

NS, Not stated.

**Table 2:** A summary of studies that have investigated the fate of antimicrobial resistant genes (ARGs) after heat treatments.

Reference	[74]	[10]	[75]	[76]
Mimic	Cooking—boiled (20 min), grilled (10 min), microwaved (5 min, 900 W), or autoclaved (20 min, 121 °C)	General heat treatments	Milk pasteurization (sterilization)	Non-food autoclaving
Medium	Chicken, beef, pork	Saline	Milk and elution buffer	Distilled water and in presence of salt
Evaluation temperatures (°C)	Not Stated	40, 50,60, 70, 80, 90, 100	63.5, 121	121, 135
Species	*E. faecalis*	*E. coli*	*S. aureus*, *S. sciuri*	Plasmid (pUC18)
Antimicrobial Resistance Genes (ARGs) present	*aac(6′)-Ie-aph(2″)-Ia*	*bla*_CTX-M-1_, *bla*_CMY-2_, *tet*A, *str*A	*blaZ*, *mecC*, *tetK*	NS
Stated antimicrobial resistance profiles	Aminoglycosides, except to streptomycin(predicted profile, not tested)	Cephalosporins, tetracycline, streptomycin	Penicillin, methicillin, tetracycline	Ampicillin
Recipient species	*E. faecalis*	*E. coli*	*S. aureus*	*E. coli*
ARGs detected post treatment from non-culturable samples	YES	YES	YES	-
Transformation demonstrated	NO	YES 70 °C for 30 min	YES 63.5 °C for 30 min	YES 121 °C for 15 min
